# Supplementary material for: Smooth Interpolating Curves with Local Control and Monotone Alternating Curvature
Source: Comput Graph Forum. 2022 Oct 6;41(5):25–38. doi: 10.1111/cgf.14600 (PMC9827861; doi:10.1111/cgf.14600)
Supplement: Supplementary file 1 — Supplement Material [file CGF-41-25-s001.zip › Local-Smooth-Interpolating-MonoCurvature/extern/clothoids/submodules/Utils/docs/api-cpp/program_listing_file_Token.cc.html]

Program Listing for File Token.cc — Utils v1.2

### Navigation

- index
- toc
- Utils »
- Program Listing for File Token.cc

# Program Listing for File Token.cc¶

↰ Return to documentation for file (`Token.cc`)

```
/*--------------------------------------------------------------------------*\
 |                                                                          |
 |  Copyright (C) 2017                                                      |
 |                                                                          |
 |         , __                 , __                                        |
 |        /|/  \               /|/  \                                       |
 |         | __/ _   ,_         | __/ _   ,_                                |
 |         |   \|/  /  |  |   | |   \|/  /  |  |   |                        |
 |         |(__/|__/   |_/ \_/|/|(__/|__/   |_/ \_/|/                       |
 |                           /|                   /|                        |
 |                           \|                   \|                        |
 |                                                                          |
 |      Enrico Bertolazzi                                                   |
 |      Dipartimento di Ingegneria Industriale                              |
 |      Universita` degli Studi di Trento                                   |
 |      email: enrico.bertolazzi@unitn.it                                   |
 |                                                                          |
\*--------------------------------------------------------------------------*/

#ifndef DOXYGEN_SHOULD_SKIP_THIS

#include "Utils.hh"

namespace Utils {

  bool
  Tokenizer::next_token() {
    size_t i = m_string.find_first_not_of( m_delimiters, m_offset );
    m_offset = m_string.length();
    if ( string::npos == i ) return false;

    size_t j = m_string.find_first_of( m_delimiters, i );
    if ( string::npos == j ) {
      m_token = m_string.substr(i);
      return true;
    }

    m_token  = m_string.substr(i, j - i);
    m_offset = j;
    return true;
  }

  void
  split_string(
    string const   & str,
    string const   & sep,
    vector<string> & arr
  ) {
    Tokenizer tk( str, sep );
    while ( tk.next_token() ) arr.push_back(tk.get_token());
  }

}

#endif
```

### Quick search

### Table of Contents

- rang - Colors for your Terminal
- Zstr
- C++ API

«
hide menu

menu
sidebar
»

### Navigation

- index
- toc
- Utils »
- Program Listing for File Token.cc

© Copyright 2021, Enrico Bertolazzi.
Created using Sphinx 4.2.0.
